# Neuropathology of 30 deceased patients with COVID-19: a case series in Tehran, Iran

**DOI:** 10.1097/MS9.0000000000000203

**Published:** 2023-02-07

**Authors:** Majid Nouri, Saeed Soleiman-Meigooni, Shadi Mohammadi, Mehdi Sakhabakhsh, Ramin Yaghmayee, Mahtab Fotoohi

**Affiliations:** a Department of Infectious Diseases, Faculty of Medicine; b Infectious Diseases Research Center; c Department of Obstetrics and Gynecology, Khanevadeh University Hospital; d Department of Neurology, Faculty of Medicine; e Department of Pathology, Khanevadeh University Hospital, Aja University of Medical Sciences, Tehran, Iran

**Keywords:** case series, neuropathology, SARS-CoV-2

## Abstract

Severe acute respiratory syndrome coronavirus 2 (SARS-CoV-2) can affect the nervous system and result in neurological symptoms. The most common feature of central nervous system involvement is hypoxia and congestion. This study aimed to evaluate the histopathology of cerebral tissue in deceased patients with coronavirus disease 2019 (COVID-19).

**Methods:**

In a case series study, we took cerebral samples of 30 deceased patients with COVID-19 through supraorbital bone from January to May 2021. The samples were fixed in a formalin solution, stained with haematoxylin–eosin dyes and studied by two expert pathologists. The Ethics Committee of AJA University of Medical Sciences approved this study with code IR.AJAUMS.REC.1399.030.

**Results:**

The mean age of the patients was 73.8 years, and the most common underlying disease was hypertension. Cerebral tissue samples showed hypoxic–ischaemic changes in 28 (93.3%), microhaemorrhage in six (20%), lymphocytic infiltration in five (16.7%) and thrombosis in three samples (10%).

**Conclusion:**

Hypoxic–ischaemic change was the most common neuropathology in our patient. Our study showed that many patients with severe COVID-19 may develop central nervous system involvement.

Highlights

- Neuropathology of 30 deceased patients with coronavirus disease 2019, the most comprehensive autopsy study in Iran.
- Presence of pathology in more than 93% of the patients.
- Presence of microhemorrhage in 20% of the patients.
- Presence of encephalitis in 16.7% of the patients.

## Introduction

Unlike the Delta wave of the coronavirus disease 2019 (COVID-19) pandemic, where most patients died due to respiratory distress caused by viral pneumonia, the mortality rate is decreasing in the Omicron wave. In contrast, the morbidity rate seems to increase by recognising new complications^1^. Long COVID syndrome is a newly defined complication of severe acute respiratory syndrome coronavirus 2 (SARS-CoV-2) infection. It may occur at any age and is characterised by fatigue, myalgia, dyspnoea, gastrointestinal, cardiac and neurological symptoms that last months after acute infection. Neurological complaints of long COVID syndrome include headache, sleep disturbance, taste and smell dysfunction and mental impairments^2^. One study showed that 57% of patients with COVID-19 developed one or more long COVID features in 6 months^3^.

Based on neuropathological studies, a wide range of cerebral tissue damage may occur during COVID-19, which may contribute to the long neurological complaint. One autopsy study reported focal haemorrhage, axonal injury, accumulation of macrophages and areas of necrosis with loss of white matter compatible with acute encephalomyelitis in a deceased patient with COVID-19^4^. Another study on four deceased patients revealed multiple microhaemorrhages and inflammatory infiltration in a cerebral microvessel consistent with endotheliitis and suggested that microhaemorrhage increased the risk of related vascular encephalopathy^5^. In an autopsy study on two patients, the neuropathological findings revealed thrombotic microangiopathy and severe multifocal cortical infarction with extensive perivascular calcification and brainstem encephalitis^6^. Our study aimed to evaluate the cerebral histopathology of a series of deceased patients with COVID-19.

## Method

This study was a single-centre prospective case series on deceased patients with COVID-19. We registered our study in Iran’s National Committee for Ethics in Biomedical Research, with the ethical code IR.AJAUMS.REC.1399.030. We performed this case series in an academic hospital in Tehran between January and May 2021. Our subjects were deceased patients with PCR-confirmed severe COVID-19 admitted into the ICU during the same period. The severity of the disease was defined as the 9th Edition of diagnosis and treatment of COVID-19 by the Iranian Ministry of Health, consisting of high-risk patients with a respiratory rate of more than 30 per minute, SpO_2_ less than 90%, PaO_2_/FIO_2_ less than 300 mmHg and more than 50% pulmonary involvement on computed tomography (https://irimc.org/news/id/45952). We obtained written informed consent from the first-degree relatives of deceased patients before the sampling. All of the selected corpuses were transferred to the dissection room of the same hospital for the sampling, 6–24 h after death. We used a 16-gauge Jamshidi needle for the sampling. We took the cerebral samples through the right or left supraorbital bone and took a 2–4 cm vermiform sample by mild suction. Both the trainee physician and assistant who performed the sampling were equipped with a face mask and goggles, a long-sleeved fluid-resistant gown and nonsterile, nitrile, latex gloves. The dissection room is also equipped with negative-pressure ventilation.

We took at least two cerebral tissue samples from each corpus. The sample tissues were placed in a container of formalin 10%, embedded with paraffin. We sent the sample tissues to the pathology laboratory of the same hospital. All tissues were stained with hematoxylin and eosin dyes after cutting by the microtome machine. Two expert pathologists studied the tissues separately, using a light microscope. We finally collected all clinical and pathological data and analysed them using the SPSS software, version 26, by Fischer’s exact test. This case series has been reported in line with the Preferred Reporting Of CasE Series in Surgery (PROCESS) Guideline^7^.

## Results

We collected 30 cerebral tissue samples from 30 patients. None of the samples failed during the sampling. Our patients included 23 males and seven females. The mean age of patients was 73.8+13.4 years, and the mean time from the onset of symptoms to death was 16.4±9.4 days. Twenty-four patients (80%) had BMI above 25 kg/m^2^. This included 17 patients (56.7%) with overweight, six patients (20%) with class 1 obesity and one patient (3.3%) with class 2 obesity, defined by the Centers for Disease Control and Prevention. The common symptoms were shortness of breath in 28 (93.3%), cough in 22 (73.3%), fatigue in 20 (66.7%), fever in 13 (43.3%) and loss of consciousness in 12 (40%). Hypertension and ischaemic heart disease were the most common underlying illnesses in 25 (83.3%) and 21(70%) patients, respectively. Other underlying diseases were diabetes mellitus in 13 (43.3%), chronic renal failure in 10 (33%) and chronic lung disease in two (6.7%) patients. Primary laboratory findings showed leukocytosis (WBC>10,000/ml) in 10 (33%), leukopenia (WBC<4000) in four (13.3%) and normal WBC in 16 (53.3%) patients. Two patients (6.7%) had a platelet count below 100×10^5^ at the time of admission. The D-dimer level was below 400 ng/ml in 14 patients (46.7%), between 400 and 800 ng/ml in nine patients (30%) and more than 800 ng/ml in seven patients (23.3%). C-reactive protein was in the normal range in two (6.7%), between 10 and 40 in seven (23.3%), 40–80 in 19 (63.3%) and more than 80 mg/l in two (6.7%) patients.

**Figure 1:**
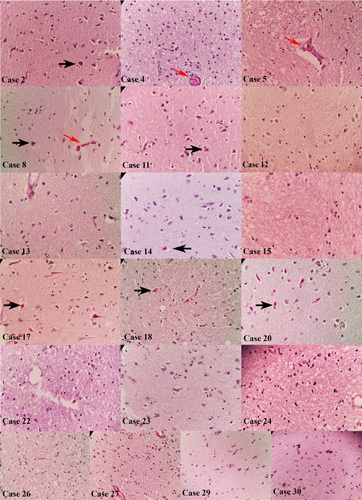
Brain tissues in cases 2, 4, 5, 8, 11, 12, 13, 14, 15, 17, 18, 20, 22, 23, 24, 26, 27, 29 and 30 showed hypoxic–ischaemic changes in all tissues, microthrombosis (red arrow), and eosinophilic neuronal degeneration (black arrow).

**Figure 2:**
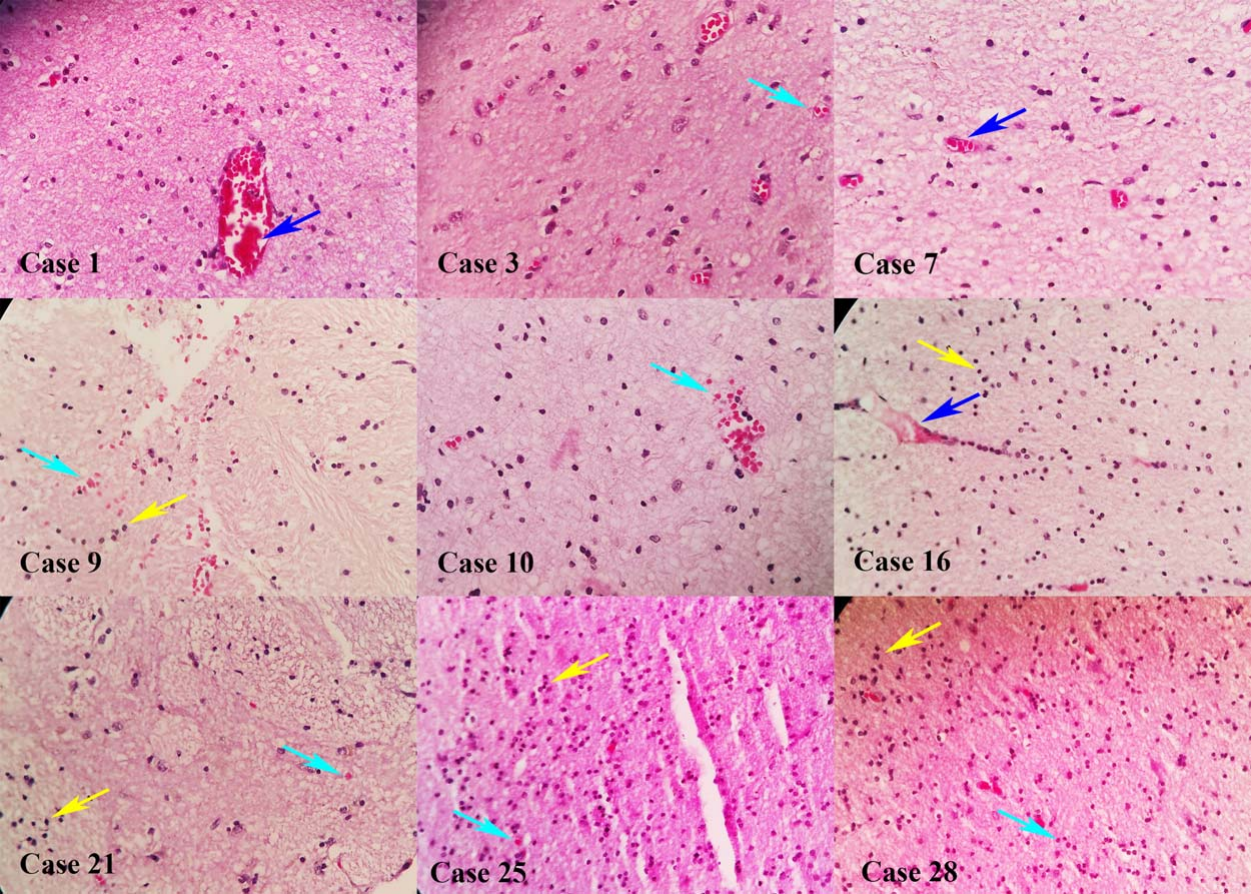
Brain tissues in cases 1, 3, 7, 9, 10, 16, 21, 25 and 28 showed dilated vessels and microvascular fibrin thrombosis (blue arrow), cerebral haemorrhage (cyan arrow) and mononuclear and polymorphonuclear inflammation (yellow arrow).

The histopathological study revealed cerebral pathology in 28 of 30 (93.3%) cerebral tissues. The most frequent neuropathological findings included hypoxic–ischaemic changes in 28 (93.3%) samples. Other findings consisted of microhaemorrhage in six (20%), lymphocytic infiltration compatible with encephalitis in five (16.7%) and thrombosis in three samples (10%) (Figs 1, 2). Two cerebral samples (6.6%) showed normal histology. We did not find any correlation between BMI and encephalitis (*P*=0.355), C-reactive protein level and encephalitis (*P*=0.185) by Fischer’s exact test.

## Discussion

Our study was the most comprehensive autopsy study of the neuropathology of COVID-19 in Iran. Our principal findings included hypoxic–ischaemic changes, haemorrhage, encephalitis and thrombosis. Other neuropathological studies on COVID-19 showed relatively similar results. In an autopsy study of 10 patients, hypoxic brain damage, perivascular infiltration of CD8-positive T-lymphocytes and microvascular damage were reported in all patients^8^. In another study of 13 patients, extensive microgliosis and infiltration of CD8-positive T-lymphocytes were found in all patients. Two patients had a diffuse hypoxic–ischaemic injury, and five had acute cerebral infarction. In that study, SARS-CoV-2 RNA was not detected in cerebral tissue^9^. One study on seven deceased patients with a mean age of 79 years revealed reactive gliosis, congestion, cortical neuron eosinophilic degeneration, and axonal disruption in six cases, oedema and haemorrhages in five, cerebral small vessel disease and Alzheimer type II glia in three and periventricular encephalitis and fibrin thrombi in one case. SARS-CoV-2 RNA was found in that study in all patients^10^. Another autopsy series of seven patients with a median age of 69.5 years showed that the virus was bound to a subset of neurovascular pericyte via ACE2 receptors. The viral genome was detected in the vascular wall, resulting in perivascular inflammation, blood–brain barrier rupture and neurological symptoms^11^. In one autopsy study, all cerebral samples showed nonspecific changes, including hypoxia and microglial activation, especially in the brainstem. But encephalitis was not found^12^.

Cerebral tissue inflammation may contribute to the development of long COVID syndrome. One study showed that damage to the blood–brain barrier and cerebral inflammation by CD8-positive T cells occurred in the perivascular space, and viral antigen was found in cerebral tissue^13^. Another study showed that microgliosis and rupture of the blood–brain barrier after SARS-CoV-2 infection might alter neurotransmission, neurogenesis and nerve damage. These changes resulted in neuropsychiatric symptoms and disorders in the patient’s learning, memory and performance^14^. A neuropathological autopsy study on eight patients revealed that infiltration of T cells in the brain parenchyma could activate microglia and astrocytes and result in cognitive function^15^. Uncommon complications may also occur following SARS-CoV-2 infection that causes neurology dysfunction. One study reported acute haemorrhagic necrotising encephalitis in a 2-month-old male infant^16^, and another reported acute necrotising encephalopathy in a pregnant woman following SARS-CoV-2 infection^17^. Table 1 compares our results with some studies with a sample size close to ours.

**Table 1:** Neuropathological findings of our study and similar studies.

Reference	No of patients	Mean age	Hypoxic–ischaemic changes (%)	Infarction (%)	Haemorrhage (%)	Encephalitis (%)	Detection of SARS-CoV-2 in cerebral tissue (%)
Matschke *et al*.^18^	43	76	86	14	0	79	53
Thakur *et al*.^19^	41	74	100	44	19	93	NA
Serrano *et al*.^20^	20	77	90	5	0	5	20
Wierzba-Bobrowicz *et al*.^21^	52	58	100	3.4	1.7	NA	NA
Fabbri *et al*.^22^	33	61	100	18.1	1.6	45.4	6
Current study	30	76	93	10	20	16.7	NA

NA, not available; SARS-CoV-2, severe acute respiratory syndrome coronavirus 2.

In association with other studies, our results showed that the most common pathological finding in SARS-CoV-2 patients is brain damage secondary to ischaemia and hypoxia. It likely occurs due to the disturbance of microcirculation. This complication may be irreversible and lead to chronic neurological symptoms. Therefore, it can also be expected that, following severe SARS-CoV-2 infection, cerebral tissue ischaemia and hypoxia result in long COVID syndrome. Also, persistent infection with SARS-CoV-2 may cause long COVID syndrome. One study found that the spike protein remains in the plasma of patients for up to 12 months after infection^23^. In contrast to the previous studies, some studies consider long COVID syndrome a psychological disorder. One study showed no abnormality in functional brain imaging in patients with long COVID syndrome^24^. We believe that alteration in cellularity and microcirculation plays an essential role in the pathology of long COVID syndrome. Other probable mechanisms are also related to long COVID syndromes, such as microvascular thrombosis and cellular apoptosis. One study revealed the role of microvascular endotheliopathy and thrombosis, and local inflammation in long COVID syndrome^25^. Another study of 80 patients with prolonged COVID syndrome in South Africa showed fibrin amyloid microclots and significant platelet pathology in all cases^26^. Our biggest limitations were the closed sampling method, which provides a limited small sample size for pathology study, and the unavailability of immunohistochemical staining of the cerebral tissues.

## Conclusion

We think that the high prevalence of brain pathology in our study is an essential finding in patients with severe COVID-19. Numerous patients who survive after severe disease may have cerebral pathology like our patients and develop neurological complications, such as long COVID syndrome. We recommend that other scientists who will study brain pathology use both commercial and immunohistochemical staining of cerebral tissues to provide more information about cerebral injuries in COVID-19. We suggest that future studies investigate the neuropathological changes in long COVID syndrome more precisely.

## Ethical approval

Aja University of Medical Sciences approved this study with the approval code IR.AJAUMS.REC.1399.030.

## Patient consent

The corresponding author took informed written consent from all of the patient’s first-degree relatives. We respected the confidentiality of information in all stages of the study and publishing of the article.

## Sources of funding

Aja University of Medical Sciences funded this study.

## Author contribution

S.S.-M. was the main author, contributing to the study design, treatment of the patients, reviewing the literature, analysing the data and writing the primary and final draft. R.Y. and M.F. were pathologists, contributing to pathological diagnosis, reviewing the literature and writing the primary draft. M.N., S.M. and M.S. were in the research team, contributing to treating the patients, reviewing the literature and writing the primary draft.

## Conflicts of interest disclosure

All authors declared that they did not have any conflicts of interest.

## Research registration unique identifying number (UIN)

1. Name of the registry: Iran’s National Committee for Ethics in Biomedical Research.

2. Unique identifying number or registration ID: IR.AJAUMS.REC.1399.030.

3. Hyperlink to your specific registration (must be publicly accessible and will be checked): https://ethics.research.ac.ir/ProposalCertificateEn.php?id=131541&Print=true&NoPrintHeader=true&NoPrintFooter=true&NoPrintPageBorder=true&LetterPrint=true

## Guarantor

S. Soleiman-Meigooni.

## Provenance and peer review

Not commissioned, externally peer-reviewed.
